# Predicted mouse peroxisome-targeted proteins and their actual subcellular locations

**DOI:** 10.1186/1471-2105-9-S12-S16

**Published:** 2008-12-12

**Authors:** Yumi Mizuno, Igor V Kurochkin, Marlis Herberth, Yasushi Okazaki, Christian Schönbach

**Affiliations:** 1 Division of Functional Genomics and Systems Medicine, Research Center for Genomic Medicine, Saitama Medical University, Hidaka, Saitama 350-1241, Japan; 2 Immunoinformatics Team, Advanced Genome Information Group, RIKEN Genomic Sciences Center, RIKEN Yokohama Institute, Yokohama, Kanagawa 230-0045, Japan; 3 RIKEN Advanced Science Institute (ASI), MetaSystems Research Team, Yokohama, Kanagawa 230-0045, Japan; 4 Cambridge Centre for Neuropsychiatric Research (CCNR), Institute of Biotechnology, University of Cambridge, Cambridge CB2 1QT, UK; 5 Division of Genomics and Genetics, School of Biological Sciences, Nanyang Technological University, Singapore 637551, Singapore

## Abstract

**Background:**

The import of most intraperoxisomal proteins is mediated by peroxisome targeting signals at their C-termini (PTS1) or N-terminal regions (PTS2). Both signals have been integrated in subcellular location prediction programs. However their present performance, particularly of PTS2-targeting did not seem fitting for large-scale screening of sequences.

**Results:**

We modified an earlier reported PTS1 screening method to identify PTS2-containing mouse candidates using a combination of computational and manual annotation. For rapid confirmation of five new PTS2- and two previously identified PTS1-containing candidates we developed the new cell line CHO-perRed which stably expresses the peroxisomal marker dsRed-PTS1. Using CHO-perRed we confirmed the peroxisomal localization of PTS1-targeted candidate Zadh2. Preliminary characterization of Zadh2 expression suggested non-PPARα mediated activation. Notably, none of the PTS2 candidates located to peroxisomes.

**Conclusion:**

In a few cases the PTS may oscillate from "silent" to "functional" depending on its surface accessibility indicating the potential for context-dependent conditional subcellular sorting. Overall, PTS2-targeting predictions are unlikely to improve without generation and integration of new experimental data from location proteomics, protein structures and quantitative Pex7 PTS2 peptide binding assays.

## Background

Peroxisomes are ubiquitous intracellular organelles that originate from the endoplasmatic reticulum [1]. Various biosynthesis and metabolic pathways including β-oxidation of very long chain fatty acids, α-oxidation of branched and straight chain fatty acids [2], plasmalogen synthesis [3], and hydrogen peroxide detoxification [4] are located in peroxisomes. Unlike mitochondria, peroxisomes lack the ability to synthesize DNA and proteins. Therefore, all peroxisomal proteins must be imported. More than 60 proteins, predominantly enzymes and peroxisomal membrane proteins are known to be sorted from the cytoplasm or endoplasmatic reticulum to peroxisomes. Apart from a peroxisomal membrane protein specific targeting signal (mPTS) [5] two types of peroxisome targeting signals, PTS1 and PTS2, mediate the peroxisomal import of proteins by peroxisome biogenesis factors 5 (Pex5) and 7 (Pex7) [6]. The majority of peroxisome-targeted proteins contain PTS1, a C-terminally located trimer [SAGCN]-[RKH]-[LIVMAF] signal that has been refined and extended to a dodecamer motif [7,8]. Less than ten peroxisomal proteins are targeted via the N-terminally located PTS2 signal [RK]-[LVQI]-X-X-[LVIHQ]-[LSGAK]-X-[HQ]-[LAF] [9]. One of the PTS2-targeted proteins phytanoyl-CoA hydroxylase is deficient in Refsum disease and rhizomelic chondrodysplasia punctata type 1. These and other inherited peroxisomal disorders caused by deficiencies in PEX proteins and ten other peroxisomal enzymes [10] have significantly contributed to the understanding of metabolic pathways in peroxisomes. However, several regulatory mechanisms including intra-peroxisomal processing of imported enzymes and their degradation, glycophospholipid metabolism, or oxidative stress defense in mammalian peroxisomes cannot be fully explained with the known set of peroxisomal proteins. In addition, the peroxisomal localization of PTS1-containing viral proteins [11] and piggy-back type targeting [12] imply that cellular proteins with and without PTS and hitherto unknown peroxisomal location maybe sorted to peroxisomes under certain conditions [13].

Attempts to identify the peroxisomal proteome from subcellular fractions of rat livers using mass spectrometry [14] led to the discovery of new peroxisomal proteins but also missed a number of known peroxisomal proteins [15]. Alternatively, new peroxisome-targeted proteins can be predicted computationally. For example PTS1Prowler is predicting whether a protein with C-terminal PTS1 sequence is targeted to peroxisome [16]. Yet, the performance of subcellular location predictions, for example PSORT II [17], pTARGET [18] or PTS1 predictor [19] is limited by the small number of peroxisomal training data compared to the data on nuclear, mitochondrial and cytoplasm-located proteins. Even the predictions for known peroxisomal proteins by different programs may not show agreement in terms of subcellular location.

We therefore developed a computational PTS1 screening method combined with manual annotation steps to identify new peroxisome candidates from protein coding sequences of GenBank^® ^[15]. The effort led to the identification of Tysnd1, a peroxisomal protease that processes PTS1 and PTS2-targeted enzymes involved in β-oxidation [20]. Encouraged by this finding we decided to apply a similar approach for identifying new PTS2-targeted proteins, while improving the first-pass experimental confirmation of remaining PTS1 and new PTS2 candidates. Unlike the PTS1 screening, regular expression and Hidden Markov Model (HMM) profile searches were used to extract PTS2-containing candidates from GenBank^®^. For rapid confirmation of peroxisomal protein candidates by confocal laser scanning microscopy we established the cell line CHO-perRed that stably expresses a peroxisome-targeted red fluorescence reporter.

Preempting the results, none of the experimentally tested PTS2 candidates localized to peroxisomes. Zinc binding alcohol dehydrogenase Zadh2, one of two tested PTS1 candidates, was confirmed and preliminary characterized. Yet, the non-peroxisomal location of PTS2 candidates raised interesting questions about differential targeting and the future of subcellular targeting predictions, including their validation.

## Methods

### Regular expression search

Putative translation of non-truncated coding sequences (CDS) of more than 99 amino acids length were retrieved from mRNA entries of GenBank^® ^(Release 146.0) primate (PRI), rodent (ROD) and high-throughput cDNA (HTC) divisions. The extracted 117,354 CDS were screened with the EMBOSS suite program [21] fuzzpro for the presence of the modified PTS2 signal [RK]-[LVQI]-{P}-x-x-[LVIHQ]-[LSGAK]-x-[HQ]-[LAF]. Proline was excluded at position three of the original PTS2 regular expression [9] because it can prevent targeting. Mismatches were not allowed and the search space was limited to the first 100 amino acids.

### HMM profile search

A HMM profile is a position-specific scoring model derived from a multiple sequence alignment. The PTS2 HMM profile was created from known functional PTS2 signals of peroxisome-targeted proteins (Additional File 1) using HMMbuild [22]. The calibrated HMM profile was used to search the CDS set sequences, containing the first 100 amino acids, with HMMER.

### Filtering and annotation of candidates

Both regular expression and HMM profile searches will yield sequences matches that are redundant, associated with known PTS2-targeted proteins, or with candidates that are unlikely to be localized to peroxisomes. To eliminate these sequences we applied a triage supported by manual curation, similar to the filtering procedure described by Kurochkin *et al*. [15]. Briefly, 36 known PTS2 containing sequences (Additional File 2) were eliminated from both regular expression (447) and HMMER search (151) results. Information on protein family classification and functional domains of the protein candidates were extracted from InterPro (release 9.0) by using InterProScan (version 8.0). BLASTP (E-value less than e-20) searches against non-redundant GenBank^® ^CDS translations were used to establish biological informative names for sequences without descriptions. Sequences that contained only motifs incompatible with peroxisomal localization (e.g., RNA-helicase (IPR0006050)), or that were supported by an unequivocal PSORT II [17] nuclear localization were eliminated. In addition, we predicted protein solubility and the numbers of trans-membrane spanning regions using the programs SOSUI [23] and TMAP [24]. Only sequences that resulted in "soluble" predictions and no more than one trans-membrane spanning region were considered.

### Cell culture and transfection

CHO-K1 Chinese hamster ovary cells (Cell Bank, RIKEN BioResource Center) were cultured in Dulbecco's modified Eagle's medium (GIBCO) containing 0.1 mM non-essential amino acid (GIBCO) and 10% (v/v) fetal bovine serum (Bio West) at 37°C in a humidified atmosphere of 5% CO_2_. One day before transfection, cells were seeded in 6-well plates and grown for one day in 2 ml medium without antibiotics. At 90–95% confluence the cells were transfected using Lipofectamine 2000 (Invitrogen) according to the manufacturer's instructions. The plasmid DNA was diluted in Opti-MEM I reduced serum medium (GIBCO). Four to six hours after transfection the medium was replaced with fresh antibiotic-free growth medium.

### Selection of stable cell line

CHO-K1 cells were transfected with pDsRed2-Peroxi (variant 2 of red fluorescent protein derived from *Discosoma sp*.), encoding the peroxisome-targeted DsRed2 reporter protein (BD Biosciences Clontech). Stably transfected CHO-K1 cells were selected with G418-supplemented medium (Nacalai Tesque) at a final concentration of 1 mg/ml. After two weeks of G418 selection, the cells were seeded in 96-well plates and monitored by fluorescence microscopy. Wells containing only red fluorescent cells were chosen for subculturing (CHO-perRed). The stable cell line CHO-perRed was maintained in the 1 mg/ml G418-containing medium to avoid the loss of the transfected DNA. After a maximum of five passages cells were frozen.

### Localization of novel peroxisomal candidates using confocal microscopy

CHO-K1 or CHO-perRed cells were plated onto glass cover slips. CHO-perRed cells were transfected with expression vectors pcDNA3.1/NT-GFP-Zadh2-TOPO, pcDNA3.1/NT-GFP-KBTBD10-TOPO, pcDNA3.1/CT-Galk2-GFP-TOPO, pcDNA3.1/CT-Qpctl-GFP-TOPO, pcDNA3.1/CT-Fut8-GFP-TOPO, pcDNA3.1/CT-Sytl3-GFP-TOPO, pcDNA3.1/CT-Ppp3ca-GFP-TOPO and pcDNA3.1/CT-Acaa1-GFP-TOPO (for construct details see Additional File 3). The PTS2 candidates (Galk2, Qpctl, Sytl3, Fut8, and Ppp3ca) and known PTS2-located thiolase (Acaa1) were expressed as fusion proteins with GFP at the C-terminus. The PTS1 candidates KBTBD10 and Zadh2 was expressed as N-terminal GFP fusion proteins. Live-cell microscopy using the 63× objective of the laser scanning confocal microscope TCS SP2 (Leica) was performed 24 h after transfection of the GFP fusion protein constructs. The laser spectra 488 nm and 543 nm were used for observation of GFP and DsRed2, respectively.

### Bezafibrate treatment, high fat diet and quantitative real-time PCR

These experiments were performed as previously described by Kurochkin *et al*. [20]. Details are given in Additional File 3.

## Results and discussion

### Peroxisomal protein candidates

The prediction of PTS2-targeted proteins from translated CDS of GenBank^® ^rodent and primate mRNA sequences using regular expression yielded 447 matches whereas the HMM profile search [21] produced 151 hits (E-value < 0.01). Of 519 total matches 79 (13.2%) were detected by both methods. Seventy-two (13.9%) matches were unique to HMMER and 368 (70.9%) matches to the regular expression searches. The results reflect the difference of PROSITE-type matching of all possible PTS2 variants, and PFAM-style position-specific scoring that corrects for unequal representation of PTS2 residues. Thirty-six sequences that were identified by both methods corresponded to known PTS2-targeted proteins encoded by six mouse genes and their eleven orthologs in human, rat, and/or guinea pig (Additional File 2).

After visual inspection of 462 new candidates, supported by results of BLASTP and InterPro motif searches as well as predictions of transmembrane-spanning regions and PSORT II subcellular localization, we excluded 435 sequences that were either redundant or contained unambiguous features, deemed incompatible with peroxisomal functions (e.g., transcription factor, histone, RNA-binding). Of the remaining 27 candidates, representing 14 mouse genes, eight human and five rat orthologs, twelve sequences showed a conserved PTS2 signal in all three species (Fut8, Ppp3ca, Ppp3cb, and 2410005O16Rik). Eight candidates carried a conserved PTS2 signal in mouse and human (Galk2, Qpctl, Sytl3, and Zmiz1). Two mouse PTS2 candidates, E330021D16Rik and 6030452D12Rik, lacked mammalian orthologs. Four candidates (Armc6, Wdr6, Adhfe1, and Pgm2) had human orthologs, but without PTS2. Details of the detection method, PTS2 signal and selection criteria for experimental testing of the 14 mouse candidates are shown in Table 1A and Additional File 4. Five candidates (Galk2, Qpctl, Sytl3, Fut8, and Ppp3ca) containing conserved PTS2 were chosen for co-localization studies. In addition, we included the PTS1 candidates (Table 1B) sarcosin (KBTBD10) and Zadh2 in the experimental evaluation. Both candidates were predicted in an earlier study [15], but have not been evaluated by us.

**Table 1:** Predicted peroxisome-targeted candidates and their experimentally determined localization. (A) PTS2-targeted candidates in mouse. (B) Mouse and human PTS1-targeted candidates taken from a previous publication [15]. RE: regular expression search with perfect match to motif; HMM: Hidden Markov Model profile search; ND: not determined. The PTS2 candidates (Galk2, Qpctl, Sytl3, Fut8, and Ppp3ca) were expressed as fusion proteins with GFP at the C-terminus. The PTS1 candidates KBTBD10 and Zadh2 were expressed as N-terminal GFP fusion proteins.

**Candidate**	**Description**	**PTS signal**	**Prediction**	**Localization by microscopy**
**(A)**	**PTS2 signal**			

Galk2	galactokinase 2	RVNIIGEHI	HMM	cytoplasm

Qpctl	glutaminyl-peptide cyclotransferase-like	KLRLVVGQL	HMM	cytoplasm

Sytl3	synaptotagmin-like protein 3-a, isoform a	KLKSHLQHL	HMM+RE	cytoplasm

Fut8	alpha-1,6-fucosyltransferase	RVRVLEEQL	HMM	intracell. organelle (Golgi)

Ppp3ca	protein phosphatase 3, catalytic subunit, alpha isoform	RVDILKAHL	HMM+RE	intracell. organelle/cytoplasm

Ppp3cb	protein phosphatase 3, catalytic subunit, beta isoform	RVDVLKNHL	HMM+RE	ND; low priority clones

Zmiz1	Zinc finger, MIZ-type containing 1	RLQCIKQHL	HMM+RE	

Armc6	armadillo repeat containing 6	RLQEVSAHL	HMM+RE	

Wdr6	WD repeat domain 6	RVQNLLGHF	HM+RE	ND; low priority clones; PTS2 absent in human orthologs

Adhfe1	Fe-containing alcohol dehydrogenase 1	RVTHLLRHL	RE	

Pgm2	phosphoglucomutase 2	KIVTVKTQA	RE	

E330021D16Rik	RIKEN cDNA E330021D16 gene	RLRIVSWHL	HMM+RE	ND; protein not expressed

6030452D12Rik	RIKEN cDNA 6030452D12 gene	RLRVIREQL	HMM	ND; no support for protein

2410005O16Rik	RIKEN cDNA 2410005O16 gene	KVEEILAQA	RE	ND; sequence conflict

**(B)**	**PTS1 signal **[15]			

Zadh2	Zinc binding alcohol dehydrogenase, domain containing 2	SKL	RE	peroxisomes

KBTBD10	kelch repeat and BTB (POZ) domain containing 10	SKL	RE	cytoplasm

### Establishment of stably transfected cell line for peroxisomal co-localization studies

**Figure 1:**
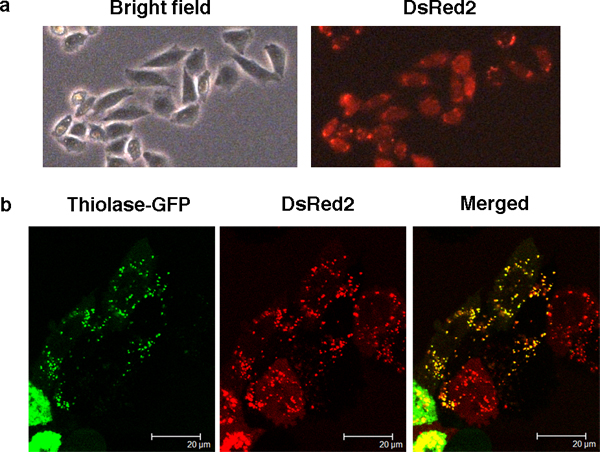
**Microscopy of CHO-perRed peroxisomal marker cells**. A. Bright field and fluorescent images of CHO-perRed cells obtained by fluorescent microscopy (original magnification, 100 ×). B. Laser scanning confocal microscopy images (original magnification, 630 ×) of GFP-fused Acaa1 (green) and DsRed2 (red). The merged images (yellow) indicate co-localization of Acaa1 and DsRed2.

A commonly used method to support computationally predicted subcellular localizations is testing cells for co-localization of the transiently transfected GFP-tagged candidate and a fluorescent marker. Variation in the copy number of integrated plasmids expressing the marker protein in transiently transfected cells, and time-consuming optimization of the ratio between GFP-fused candidate and the marker DNA for each experiment render this method unsuitable for systematic testing of larger number of candidates with minimum variation in experimental conditions. Since CHO-K1 cells are widely used in co-localization experiments of peroxisomal proteins, we stably transfected the peroxisomal marker dsRed2-PTS1 into CHO-K1 cells to establish the cell line CHO-perRed (Figure 1).

The morphology of CHO-perRed cells was comparable that of CHO-K1. DsRed2 was uniformly expressed in almost all cells (Figure 1A). After five passages the expression of DsRed2 has not changed (Additional File 5). Co-localization of known peroxisomal proteins with DsRed2 was tested by transfecting CHO-perRed cells with GFP-fused Acca1 (thiolase), which is targeted by PTS2. Confocal microscopy confirmed the overlapping punctuate distribution of Acaa1 and DsRed2 (Figure 1B, merged) that is characteristic for peroxisomal localization.

### Peroxisomal localization of PTS1-containing candidate Zadh2 in CHO-perRed cells

**Figure 2:**
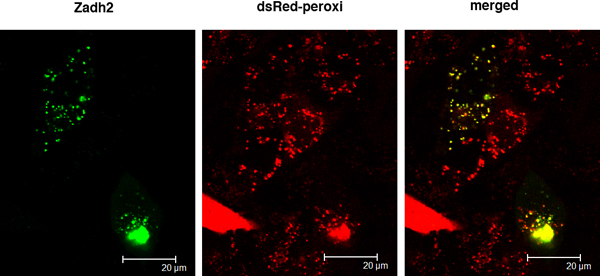
**Subcellular localization of Zadh2 protein**. GFP-fused Zadh2 transfected CHO-perRed cells were analyzed by laser scanning confocal microscopy (original magnification 630 ×). Co-localization of GFP-fused Zadh2 (green) and DsRed2 (red) is supported by the merged images (yellow).

Mouse Zadh2 co-localized with DsRed in CHO-perRed cells (Figure 2 and Table 1). The peroxisomal localization of Zadh2 was corroborated by the findings of Islinger *et al. *[25]. The authors identified Zadh2 in liver peroxisome subfractions of bezafibrate-treated rats using mass spectrometry. The peroxisomal localization of Zadh2 was demonstrated in a stably GFP-PTS1 transfected CHO cell clone. However, the function of this new peroxisomal enzyme was not further characterized.

Since computational inferred evidence from sequence analysis suggested alcoholdeydrogenase and antioxidant functions, rather than direct involvement in β-oxidation we tested whether Zadh2 is inducible by bezafibrate or high fat diet. Bezafibrate is a peroxisome proliferator activated receptor alpha (PPARα) agonist that increases liver β-oxidation of fatty acids. Zadh2 expression upon bezafibrate treatment was slightly reduced (0.74 ± 0.06, p = 0.04407) compared to normal-diet controls (Additional File 6), indicating a non-PPARα related activation mechanism. In accordance with previously published data [20], bezafibrate significantly induced thiolase expression (6.28 ± 0.93, p = 0.00087) whereas Scp2 expression remained unchanged. On the other hand, high fat diet induced Zadh2 expression (1.84 ± 0.24, p = 0.00697) while the expression β-oxidation enzyme thiolase remained more or less at control levels. Considering that the activity of alcohol dehydrogenase is not affected by bezafibrate [26], Zadh2 may indeed assume protective functions against peroxisomal peroxidation of polyunsaturated fatty acids. Whether Zadh2 is a functional homolog of the cytosolic NADP-dependent quinone oxidoreductase [15], which also belongs to the superfamily of Zn-containing alcohol dehydrogenase remains to be established in future studies.

### Non-peroxisomal localization of PTS2- and PTS1-containing candidates in CHO-perRed cells

The predicted subcellular locations of one PTS1 and five PTS2 candidates did not conform to the observed localization in CHO-perRed cells (Table 1 and Additional File 7). KBTBD10, Galk2, Sytl3 and Qcptl were detected in the cytoplasm. Fut8 localized to spotted structures in the cytoplasm within proximity of the nucleus. Considering that Fut8 was initially isolated from Golgi-rich fractions of rat liver [27] the observed localization is believed to be the Golgi apparatus. PPP3ca was detected in aggregated structures in the cytoplasm with negligible punctuate localization to peroxisomes. The aggregates were reminiscent of the calcineurin clusters in COS7 cells [28].

From a computational point of view the five PTS2 candidates were false positives. Typically, the result would be indicative of poor accuracy of the initial PTS searches and/or problems in the candidate selection strategy. The initial PTS2 regular expression search used an improved motif [RK]-[LVQI]-{P}-x-[LVIHQ]-[LSGAK]-x-[HQ]-[LAF] that is much more restrictive than the original PTS2 motif [RK]-[LVIQ]-x-x-x-x-x-[HQ]-[LAF] [29]. Similarly, when building the HMM model we excluded *A. thaliana *AT1G04710 (acetyl-CoA C-acyltransferase), to obtain a tighter model. Consequently, the number of hits for both regular expression (447) and HMMER (151) without redundancy reduction was relative low and included 36 known PTS2 sequences.

The selection strategy was based on the knowledge of peroxisome biology at the time the study was planned. Since the intermingling of computational search with semi-manual candidate filtering and manual prioritization (see also Additional File 4) for experimental validation does not allow standard performance comparisons with other methods, we only used the sequences of the selected candidates, known PTS sequences and actual subcellular locations for comparisons. A complete assessment of the selection strategy would require not only the replication of all steps for the initial prediction results of other methods, but also the comparison of different semi-manual filtering steps, and the prioritization step by independent biologists. Despite the partial nature of comparisons with other methods, the emerging discrepancies and limitations of peroxisome targeting prediction approaches, including ours, may be used to re-evaluate and improve peroxisome targeting prediction strategies, particularly for PTS2.

### Comparison of subcellular locations with predictions by PTS2 Predictor

PTS2 Target Signal Predictor of PeroxisomeDB [30], which uses Blimps position-specific scoring matrix search [31], detected putative PTS2 in all candidates at the same positions as HMMER, albeit different E-values (Additional File 4). The E-values (e1-04 to e1-05) of potential PTS2 in Ppp3ca, Ppp3cb, Zmiz1, Armc6, and Wdr6 sequences were one to two magnitudes lower than HMMER E-values (~e1-03). Assessment of the predictions with published, experimentally determined subcellular locations showed that none of the proteins were reported to locate to peroxisomes (see last column of Additional Table 8A). On the other hand, the PTS2 Target Signal Predictor E-values of Galk (0.029) and Qpctl (0.013) and Fut8 (0.0055) were above the cut-off threshold of 0.005. Although the comparison with PTS2 Targeting Predictor was restricted to our candidate set, both prediction methods do not appear to be very effective.

### Comparison of PTS2 predictions with LOCATE subcellular location summaries

Comparison of the predicted peroxisomal location with available subcellular location summaries of LOCATE database [32] showed no concordance (Additional File 8A). The LOCATE subcellular location summary is based on evidence from primary literature, original experiments, and annotations derived from various databases (e.g., UniProt, SwissProt, HPRD, etc.), if available. All known PTS2-targeted mouse proteins, except Acaa1b, and four out of ten PTS2 candidates had a LOCATE summary. The summaries of Fut8, Ppp3ca, Ppp3cb and Zmiz1 displayed non-peroxisomal locations, which are in line with our experimental results (Fut8 and Ppp3ca) or literature evidence (Ppp3cb and Zmiz1).

### Variation of predicted subcellular locations across different prediction tools

LOCATE entries are also linked to integrated results of computationally more sophisticated subcellular location prediction methods CELLO, pTarget, Proteome Analyst, WoLFPSORT, and MultiLoc. When comparing the predicted subcellular locations of entries corresponding to six known PTS2 targeted proteins as well as our candidates, we noticed little agreement in the output of each prediction method (Additional File 8A). In fairness to the five methods, it should be noted that they were not specifically designed to predict peroxisomal locations. A performance comparison by Sprenger et al. [33] demonstrated an overall good performance for frequently represented locations e.g., nuclear, cytoplasmic, extracellular and mitochondrial. CELLO, MultiLoc and WoLF PSORT showed reduced sensitivity for under-represented locations, such as peroxisomes.

Interestingly, pTarget [18] and CELLO [34] predicted five of six sequences of known mammalian PTS2 targeted proteins correctly. WoLFPSORT [35] predicted Pex11c, Phyh, Acaa1 and Acaa1b as mitochondrial, Apgt as cytoplasmic and Mvk as extracelluar. Proteome Analyst [36] reported two peroxisomal (Phyh), one cytoplasmic (Mvk), two mitochondrial (Acaa1, Acca1b) and one unassigned locations (Pex11c). MultiLoc [37] is not trained to recognize PTS2. The predictions of the PTS2-containing but non-peroxisomal located proteins coincided with experimentally reported locations for Galk2, Wdr6, Ppp3ca and Pgm2, but varied for the other tested sequences.

Similarly, the recently identified PTS1-targeted Tynsd1, Zadh2 of this study and three candidates, KBTBD10, Gab2 and Scarb2, were predicted by all five programs to locate to various non-peroxisomal locations. LOCATE subcellular location summaries were not available for these proteins.

When using the dedicated PTS1 prediction methods of PTS1Prowler [16] and PTS1Predictor [19] the concurrence of results with experimentally determined locations improved, particularly for PTS1Prowler. Details are shown in Additional File 8B. Tysnd1 and Zadh2 were correctly predicted as PTS1 targeted with scores of 0.98 and 0.99 respectively. Since PTS1prowler is an add-on to the general subcellular location predictor PProwler [16], prediction results indicated also a possible mitochondrial location for Tysnd1 (score = 0.76). Zadh2 was given a score (0.96), almost equal to the peroxisomal location score (0.99), for an unspecified other subcellular location. These predictions contrast independent experimental results.

PTS1Predictor predicted all five proteins as peroxisome-targeted, but with varying scores and false positive probabilities (Additional File 8B). The peroxisome-targeted Tysnd1 received the highest score (16.264) and lowest false positive probability followed by Gab2 (8.628), Scrab (3.626) and KBTBD1 (2.734) which are actually non-peroxisomal. The peroxisomal Zadh1 had the lowest score (2.052).

Another three PTS1 prediction tools are PeroxiP [34] and PeroxisomeDB PTS1 Target Signal Predictor [30]. PeroxiP could not be evaluated due to web server problems at the time of preparing the manuscript. The Target Signal Predictor for PTS1 is using a twelve amino acid PTS1 position-specific scoring matrix for the Blimps search [28]. Since the Blimps search output provides hits over the entire sequence length rather than the C-terminus were PTS1 resides, the results require manual inspection by the user.

### Concept of oscillating PTS

The unsatisfactory outcome of the predictions suggests that sequence-based features and evolutionary conservation, even in combination with manual annotation and human-inferred supporting associations (e.g., Galk2 and Ppp3ca; Additional File 4) are insufficient for predicting the correct subcellular location, particularly for PTS2-containing candidates. Similar observations were made by Neuberger *et al*. [13] for 23 vertebrate lysozyme sequences containing a computational *bona fide *PTS1 sequence that causes PTS1Predictor [19] to classify them as peroxisome-targeted candidates. In fact, lysozymes are secreted proteins. When the N-terminal signal peptide was replaced by a GFP tag, lysozyme was targeted to peroxisomes. Since PTS1 became functional upon replacement of the signal for secretion it was dubbed a "silent PTS", rather than a false positive [13]. We believe that the existing disagreement about the cytosolic and peroxisomal locations of the GMPH kinase family members mevalonate kinase [39-41] and phosphomevalonate kinase [42,43] might be rooted in PTSs that oscillate from "silent" to "functional", depending on their surface accessibility. The localization of the third GMPH kinase family member Galk2 in this study may underlie the same mechanism.

The recently characterized peroxisomal matrix protein, human soluble epoxide hydrolase (EPHX2) lends support to our hypothesis. EPHX2 contains a rare PTS1 signal (SKM) and two PTS2 signals [44]. It locates to the cytoplasm or peroxisome depending on the surface accessibility of PTS1 and protein expression level. The two PTS2 signals are non-functional due to surface inaccessibility. *In vivo *data showed that the PTS1 signal poorly mediated peroxisomal targeting if the EPHX2 protein expression level was low. According to the authors, 90% of EPHX2 of was found in the cytoplasm. On the other hand, increase of EPXH2 PTS1 surface accessibility led to peroxisomal import that was independent of the protein expression level.

The concept of oscillating PTS should not be misunderstood as excuse for the poor performance of PTS2 predictions. Instead, it highlights the need to build PTS1 and PTS2 predictors that can simulate the selection criteria of Pex5 and Pex7 receptors. The comparison of free energy differences of PTS1 peptide Pex5 receptor binding showed mediocre correlation (0.44) with the PTS1Predictor scoring results [45]. Improvements in predicting PTS2 targeting are expected to come from the integration of PTS2 accessibility information gleaned from three-dimensional structures of PTS2-containing proteins and quantitative measurements of PTS2 peptide affinities to Pex7. Improved prediction indicators may not entirely solve the problem of differential subcellular sorting and interactions with cytosolic proteins or even other organelles.

### Differential subcellular sorting and context dependency

For two peroxisomal enzymes, mammalian alanine-glyoxylate aminotransferase (AGT) [46-48] and yeast catalase A (CTA1) [49] differential targeting has been associated with changes in dietary or nutrient conditions. AGT has a N-terminal mitochondrial targeting signal (MTS) and a C-terminal PTS1. The dual-targeting capacity of AGT to locate preferentially to the peroxisomes (herbivors) or mitochondria (carnivors) is likely correlated with the shifting diet in the evolution of carnivors [46,47]. In case of human AGT PTS1 targeting is further complicated by the dependency on an ancillary 22 amino acid peroxisomal signal [48]. Analyses of AGT substitution ratios in the MTS of primates [50] and Carnivora [46] indicated that positive selection pressure by an increase of herbivorous diet component in primates affected the N-terminal MTS and use of two neighbouring in-frame translation initiation sites. The outcome is seen in a decreased or abolished mitochondrial targeting capacity.

Indeed, statistical analysis of across 77 mammalian species demonstrated that the localization significantly correlated with diet rather than phylogeny [47]. For example, in human, rabbit, and guinea pig AGT localises to the peroxisomes, whereas in dog and cat, AGT is predominantly mitochondrial. In rat, AGT localises to both, peroxisomes and mitochondria. Subcellular localisation testing of peroxisom-targeted enzymes over a wider range of species may reveal more cases of diet context-dependent differences in targeting.

Even more striking is the nutrient-dependent differential targeting of CTA1 in yeast [49]. CTA1, which has both PTS1 and PTS2 signals but no mitochondrial targeting signal, localizes almost exclusively to the mitochondria when grown on raffinose under respiratory conditions. If the yeast is grown on oleate or on glucose under fermentation conditions, CTA1 is targeted to both peroxisomes and mitochondria. Details of the molecular mechanism and regulatory network are not known.

At last, the discovery of mitochondria-derived vesicles (MDVs) [51] added yet another twist to the prediction of subcellular protein locations. MDVs were shown to transport various mitochondrial proteins to peroxisomes where they fuse with a subset, estimated to make up around 10% of the peroxisomes [51,52]. Thus, actual mitochondria-targeted (or predicted) proteins may localize in part via MDVs to peroxisomes.

Since the aforementioned findings complicate the peroxisomal targeting prediction, more data on the structure of PTS-containing proteins and dynamics of peroxisome targeting process are needed to derive better prediction models. Dynamic changes in protein location depending on the cell conditions can be captured by location proteomics [53]. The genome-based central dogma (CD) tagging approach [54] which does not affect endogenous regulatory sequences combined with high throughput microscopy movies successfully detected previously unknown cell cycle-dependent localization changes of nuclear proteins. The submission and collation of peroxisomal data into growing integrated protein and genetic interaction resources such as BioGRID [55] may help in the evaluation of false positives/negatives and improve our understanding of protein sorting to peroxisomal and other subcellular locations.

## Conclusion

The non-peroxisomal localization of all tested PTS2-targeted candidates in CHO-perRed demonstrated the underperformance of computer-aided predictions of PTS2-containing sequences. At least PTS2 prediction methods, including ours will not improve by simply amending algorithms. New experimental data, derived from location proteomics, protein structure analysis, and quantitative PTS2 peptide Pex7 binding assays are required to lift the performance of predictions. In a few cases, the presence of a silent PTS2 indicated the potential for conditional subcellular sorting which might be worthwhile to follow up. The surface accessibility and expression context-dependent PTS1-targeting of EHXP2 [40] is an encouraging example. As CHO cells are considered a model for mammalian peroxisome research, the stable cell line CHO-perRed is expected to become an effective tool for peroxisomal location research. In view of the growing importance of subcellular localization we suggest to promote the development of other peroxisomal marker cell lines that cover cells of different ontology in parallel to community-agreed computational and experimental standards for peroxisomal localization.

## List of abbreviations used

PTS1: peroxisomal targeting signal 1; PTS2: peroxisomal targeting signal 1; CHO-perRed: Chinese hamster ovary K1 cells stably expressing variant 2 of red fluorescent protein derived from *Discosoma sp*.; PPARα: peroxisome proliferator activated receptor alpha; Zadh2: zinc binding alcohol dehydrogenase 2; Mvk: mevalonate kinase; Galk2: galactokinase 2

## Competing interests

The authors declare that they have no competing interests.

## Authors' contributions

YM and IVK performed all experiments. MH, IVK and CS predicted and annotated the PTS candidates. YM, CS and YO designed the study. CS, YM and YO wrote the manuscript. All authors read and approved the final version of the manuscript.

## Supplementary Material

Additional file 1 Sequence sources used to construct the PTS2 HMM profile. Click here for file

Additional file 2 Sequence sources of known mouse PTS2-targeted proteins and their orthologs. Click here for file

Additional file 3 Supplementary materials and methods. Click here for file

Additional file 4 Details of regular expression and HMMER profile search-derived mouse PTS2 candidates after triage and annotation plus comparison with PeroxisomeDB PTS2 Predictor Blimps Search. Click here for file

Additional file 5 CHO-perRed after two and five passages. Click here for file

Additional file 6 Measurement of Zadh2 liver mRNA levels by using quantitative real-time PCR. Click here for file

Additional file 7 Non-peroxisomal localization of five PTS2- and one predicted PTS1-containing candidates in CHO-perRed cells. Click here for file

Additional file 8 **Comparison of predicted subcellular locations with experimental data for both known and hitherto unknown PTS1 and PTS2-targeted proteins**. **A**. Comparison of LOCATE summaries and integrated prediction results with experimentally supported localization of proteins. **B**. Comparison of PTS1Prowler/PProwler and PTS1 Predictors predictions with experimentally supported localization of proteins. Click here for file
