# Supplementary material for: Predicted mouse peroxisome-targeted proteins and their actual subcellular locations
Source: BMC Bioinformatics. 2008 Dec 12;9(Suppl 12):S16. doi: 10.1186/1471-2105-9-S12-S16 (PMC2638156; doi:10.1186/1471-2105-9-S12-S16)
Supplement: Additional file 1 — Sequence sources used to construct the PTS2 HMM profile. [file 1471-2105-9-S12-S16-S1.pdf]

Additional file 1 – Sequence sources used to construct the PTS2 HMM profile

Gene	Species	Name	Accession	PTS2 signal
Phyh	Mouse	Phytanoyl-CoA hydroxylase [1]	AF023463	RLQVLLGHL
Phyp	Rat		AF121345	
PHYH	Human		BC029512	RLQIVLGHL
Pex11c	Mouse	Peroxisomal biogenesis factor 11c [2]	AK007582	RLLVVSAQF
PEX11G	Human	Peroxisomal biogenesis factor 11 gamma [3]	BC008780	RLLVVSTQL
Mvk	Mouse	Mevalonate kinase [4-6]	BC005606	KVILHGEHA
Mvk	Rat		M29472	
MVK	Human		M88468	
Acaa1b	Mouse	Acetyl-Coenzyme A acyltransferase 1B (thiolase B) [7,8]	AY273812	RLQVVLGHL
Acaa1b	Rat		J02749	
Acaa1a	Mouse	Acetyl-Coenzyme A acyltransferase 1A (thiolase A) [7,8]	AK143187	
ACAA1	Human		BC000635	
Acaa1	Rat		M32801	
POT1	Yeast	3-ketoacyl-CoA thiolase [9]	Z38059	RLQSIKDHL
Agps	Mouse	Alkylglycerone phosphate synthase [10]	BC063086	RLRVLSGHL
Agps	Rat		AF121052	
Agps	Guinea pig		Y08826	
AGPS	Human		AY544121	

Sequences in column “PTS2 signal” were used to build the HMM profile. References 1-4 and 6-10 support the PTS2-dependent import of proteins into the peroxisomes. Here, Mvk was accepted as a PTS2-targeted protein, although reports differ between cytoplasmic [5] and peroxisomal localisation [4, 6] depending on the cell line and methods used.

References

1. Jansen GA, Ofman R, Denis S, Ferdinandusse S, Hogenhout EM, Jakobs C, Wanders, RJ: **Phytanoyl-CoA hydroxylase from rat liver. Protein purification and cDNA cloning with implications for the subcellular localization of phytanic acid alpha-oxidation**. *J Lipid Res.* 1999, 40: 2244-2254.

2. Li X, Baumgart E, Dong GX, Morrell JC, Jimenez-Sanchez G, Valle D, Smith KD, Gould SJ: **PEX11alpha is required for peroxisome proliferation in response to 4-phenylbutyrate but is dispensable for peroxisome proliferator-activated receptor alpha-mediated peroxisome proliferation**. *Mol Cell Biol* 2002, 22: 8226-8240.
3. Tanaka A, Okumoto K, Fujiki Y: **cDNA cloning and characterization of the third isoform of human peroxin Pex11p**. Biochem Biophys Res Commun. 2003, 300: 819-823.
4. Biardi L, Sreedhar A, Zokaei A, Vartak NB, Bozeat RL, Shackelford JE, Keller GA, Krisans SK: **Mevalonate kinase is predominantly localized in peroxisomes and is defective in patients with peroxisome deficiency disorders**. *J Biol Chem*. 1994, 269: 1197-1205.
5. Hogenboom S, Tuyp JJ, Espeel M, Koster J, Wanders RJ, Waterham, HR: **Mevalonate kinase is a cytosolic enzyme in humans**. *J Cell Sci* 2004, 117: 631-639.
6. Kovacs WJ, Tape KN, Shackelford, JE, Duan X, Kasumov T, Kelleher JK, Brunengraber H, Krisans SK: **Localization of the pre-squalene segment of the isoprenoid biosynthetic pathway in mammalian peroxisomes**. *Histochem Cell Biol.* 2007, 127: 273-290.
7. Miyazawa S, Osumi T, Hashimoto T: **The presence of a new 3-oxoacyl-CoA thiolase in rat liver peroxisomes**. *Eur J Biochem.* 1980, 103: 589-596.
8. Swinkels BW, Gould SJ, Bodnar AG, Rachubinski RA, Subramani S: **A novel, cleavable peroxisomal targeting signal at the amino-terminus of the rat 3-ketoacyl-CoA thiolase**. *EMBO J.* 1991, 10: 3255-3262.
9. Glover JR, Andrews DW, Rachubinski RA: **Saccharomyces cerevisiae peroxisomal thiolase is imported as a dimer**. *Proc Natl Acad Sci U S A* 1994, 91: 10541-10545.
10. Biermann J and van den Bosch H: **In vitro processing of the human alkyl-dihydroxyacetonephosphate synthase precursor**. *Arch Biochem Biophys*. 1999, 368: 139-146.
