# Supplementary material for: Predicted mouse peroxisome-targeted proteins and their actual subcellular locations
Source: BMC Bioinformatics. 2008 Dec 12;9(Suppl 12):S16. doi: 10.1186/1471-2105-9-S12-S16 (PMC2638156; doi:10.1186/1471-2105-9-S12-S16)
Supplement: Additional file 2 — Sequence sources of known mouse PTS2-targeted proteins and their orthologs. [file 1471-2105-9-S12-S16-S2.pdf]

Additional file 2 – Sequence sources of known mouse PTS2-targeted proteins and their orthologs

Gene	Species	GenBank accession	PTS2	Reference
Phyh	Mouse	**AF023463**, AK168648, AF023463, D88670, BC002018	RLQVLLGHL	[1]
Phyp	Rat	**AF121345**		
PHYH	Human	**BC029512** ,AF023462, AF112977, CR542055	RLQIVLGHL	
Pex11c	Mouse	**AK007582**	RLLVVSAQF	[2,3]
PEX11G	Human	**BC008780**	RLLVVSTQL	
Mvk	Mouse	**BC005606**, AK149630**,** AF137598	KVILHGEHA	[4,5,6]
Mvk	Rat	**M29472**		
MVK	Human	**M88468**		
Acaa1b	Mouse	**AY273812**	RLQVVLGHL	[7,8]
Acaa1b	Rat	**J02749**		
Acaa1a	Mouse	**AK143187**, AY273811**,** BC012400		[7,8]
ACAA1	Human	**BC000635**, X14813, BC014474, X12966, BC011977		
Acaa1	Rat	**M32801**, BC089821		
Agps	Mouse	**BC06308**, AK031049	RLRVLSGHL	[9]
Agps	Rat	**AF121052**		
Agps	Guinea pig	**Y08826**		
AGPS	Human	**AY544121**, BC141820**,** Y09443		

Phyh: phytanoyl-CoA hydroxylase; *Pex11c*: peroxisomal biogenesis factor 11c; *PEX11G*: peroxisomal biogenesis factor 11 gamma; *Mvk*: mevalonate kinase; *Acaa1b*: acetyl-Coenzyme A acyltransferase 1B (thiolase B); *Acaa1*: acetyl-Coenzyme A acyltransferase 1A (thiolase A); *Agps*: alkylglycerone phosphate synthase.

Thirty-six sequences are considered to represent known PTS2-targeted proteins. 17 sequences (accession in bold) are encoded by six mouse genes five human, five rat and one guinea pig ortholog. These sequences were also used to construct the PTS2 HMM profile (see Additional File 7). The remaining 19 sequences are either redundant or variants (e.g., substitution of one amino acid). Known mouse PTS2-targeted proteins are encoded by *Phyh*, *Pex11c*, *Acaa1*, *Acaa1b*, and *Agps*. References 1-3 and 7-9 support the PTS2-dependent import of proteins encoded by the above

genes into the peroxisomes. Here, Mvk was accepted as a PTS2-targeted protein, although reports differ between cytoplasmic and peroxisomal localisation depending on the cell line and methods used (see also references 4-6). Orthologous sequences of mouse Phyh and Mvk were available found for rat and human. *PEX11G* is the human ortholog of mouse *Pex11c*. The sequence of a rat ortholog was not available at the time of analysis. Acaa1 orthologs were identified for rat and human. Acaa1b orthologs were only found in rat. Rodents possess three thiolases: Acaa1a, Acaa1b and Scp2. Acaa1a and Acaa1b are targeted by PTS2. Scp2 is targeted by PTS1. Human and other mammals have only one thiolase (Acaa1). Orthologs of mouse Agps were present in human, rat and guinea pig.

References

1. Jansen GA, Ofman R, Denis S, Ferdinandusse S, Hogenhout EM, Jakobs C, Wanders, RJ: **Phytanoyl-CoA hydroxylase from rat liver. Protein purification and cDNA cloning with implications for the subcellular localization of phytanic acid alpha-oxidation**. *J Lipid Res.* 1999, 40: 2244-2254.
2. Li X, Baumgart E, Dong GX, Morrell JC, Jimenez-Sanchez G, Valle D, Smith KD, Gould SJ: **PEX11alpha is required for peroxisome proliferation in response to 4-phenylbutyrate but is dispensable for peroxisome proliferator-activated receptor alpha-mediated peroxisome proliferation**. *Mol Cell Biol* 2002, 22: 8226-8240.
3. Tanaka A, Okumoto K, Fujiki Y: **cDNA cloning and characterization of the third isoform of human peroxin Pex11p**. Biochem Biophys Res Commun. 2003, 300: 819-823.
4. Biardi L, Sreedhar A, Zokaei A, Vartak NB, Bozeat RL, Shackelford JE, Keller GA, Krisans SK: **Mevalonate kinase is predominantly localized in peroxisomes and is defective in patients with peroxisome deficiency disorders**. *J Biol Chem.* 1994, 269: 1197-1205.
5. Hogenboom S, Tuyp JJ, Espeel M, Koster J, Wanders RJ, Waterham, HR: **Mevalonate kinase is a cytosolic enzyme in humans**. *J Cell Sci* 2004, 117: 631-639.

6. Kovacs WJ, Tape KN, Shackelford, JE, Duan X, Kasumov T, Kelleher JK, Brunengraber H, Krisans SK: **Localization of the pre-squalene segment of the isoprenoid biosynthetic pathway in mammalian peroxisomes**. *Histochem Cell Biol.* 2007, 127: 273-290.
7. Miyazawa S, Osumi T, Hashimoto T: **The presence of a new 3-oxoacyl-CoA thiolase in rat liver peroxisomes**. *Eur J Biochem.* 1980, 103: 589-596.
8. Swinkels BW, Gould SJ, Bodnar AG, Rachubinski RA, Subramani S: **A novel, cleavable peroxisomal targeting signal at the amino-terminus of the rat 3-ketoacyl-CoA thiolase**. *EMBO J*. 1991, 10: 3255-3262.
9. Biermann J and van den Bosch H: **In vitro processing of the human alkyl-dihydroxyacetonephosphate synthase precursor**. *Arch Biochem Biophys*. 1999, 368: 139-146.
