# Supplementary material for: Predicted mouse peroxisome-targeted proteins and their actual subcellular locations
Source: BMC Bioinformatics. 2008 Dec 12;9(Suppl 12):S16. doi: 10.1186/1471-2105-9-S12-S16 (PMC2638156; doi:10.1186/1471-2105-9-S12-S16)
Supplement: Additional file 3 — Supplementary materials and methods. [file 1471-2105-9-S12-S16-S3.pdf]

Additional file 3 - Supplementary Materials and Methods

Plasmid constructs

cDNA clones for Galk2 were amplified from mouse liver total RNA. Zadh2, Qpctl, Ppp3ca and Fut8 cDNA clones were amplified from the RIKEN Mouse Genome Encyclopedia DNA Book (K.K. DNAFORM). The mouse Sylt3 (Slp3-a) cDNA clone was kindly provided by Dr. Mitsunori Fukuda (Department of Developmental Biology and Neurosciences, Graduate School of Life Sciences, Tohoku University, Japan). KBTBD10 was amplified from human whole brain Marathon-Ready cDNA (BD Biosciences, Clontech). The PCR products of PTS2 candidates (KBTBD10, Galk2, Qpctl, Sytl3, Fut8, and Ppp3ca) and the known PTS2-targeted thiolase A (Acaa1) were subcloned into the green fluorescence protein (GFP) vector pcDNA3.1/CT-GFP-TOPO. Expression of the target as C-terminal GFP fusion protein does not interfere with function of the PTS2 signal at the N-terminus. The PTS1 candidate Zadh2 was subcloned into pcDNA3.1/NT-GFP-TOPO (Invitrogen) which expresses a fusion protein that harbors GFP at the N-terminus and PTS1 at the C-terminus. Prior to transfection all plasmid constructs were checked for the correct orientation of inserts. The PCR primers are shown below.

Gene	Forward primer sequence	Reverse primer sequence
Zadh2	ATGCTGAGGCTGGCGGCCG	TCACAGCTTACTGCTGACAGGGTGT
KBTBD10	ATGGATTCCCAGCGGGAGCTTGC	TTGTCACCTCCTTTATAGTTTAGACAGTTTAAAC
Galk2	AACATGCCCGACCTATCTGAAAGATTG	CCTCACGGAAAACCAAGGCCC
Qpctl	ACCATGAGTCCCGGGAGCCG	GTCCCAGGTACTCGGCCAGGAA
Sytl3	GAAATGGCCCACGAAGTGGACCT	GCAGGACGAGGGTCATGTCTGT
Fut8	AAAATGCGGGCATGGACTGGTTCC	TTTCAGCTTCAGGATATGTGGGATACTTGA
Ppp3ca	GAGATGTCCGAGCCCAAGGCGA	GGATATTGCTGCTATTACTGCCATTGCTG
Acaa1	GCGATGCATCGGCTGCAGGTAG	CAAAGACCGCAGCAGCTCCCATC

Bezafibrate treatment and high fat diet

Six Male C57BL/6J mice of five weeks age were fed for two weeks with CE2 rodent. After two weeks three mice were switched for three weeks to 0.5 bezafibrate (v/w) supplemented rodent chow. Three C57BL/6J control mice were maintained for five weeks on a standard rodent chow diet. Another three C57BL/6J mice were fed for five weeks with QuickFat (CLEA Japan) high-fat diet. After five weeks all mice were sacrificed and liver RNA isolated for quantitative real-time PCR.

Quantitative real-time PCR

Mice livers were stabilized in RNA-later solution (Takara) and total RNA isolated using RNeasy Miniprep kit (Qiagen). An aliquot of 500ng total RNA was reverse transcribed with BioScript First Strand cDNA synthesis kit (Bio Script) using Oligo dT 12-18mer primers.

Real-time PCR was carried out in a reaction mixture containing 1x Power SYBER Green PCR Master Mix (Applied Biosystems), 100nM of each primer (see below), and cDNA obtained from 20ng of total RNA. Real-time PCR conditions were 10 min at 95°C followed by 40 cycles of 30 seconds at 95°C, 1min at 55°C and 30 seconds at 72 °C. Results were normalized to *Gadph* control mRNA levels. DNA primers specific for *Zadh2*, *Scp2*, *Acaa1* and *Gadph* (control) were designed to produce a maximum amplicon length of 189 bp.

**Genes**	**Forward primer sequence**	**Reverse primer sequence**
Zadh2	TGAGCCCTAACTTCCACGAG	ATGTCAAAGGGTGGCTTCAG
Acaa1	TCCAGTCAGGTGAGTGATGG	ATTCACAGTCAGCCCTGCTT
Scp2	CCGGAAAGAGGCAGGTTC	AAAGACGAGGTTTGCCTTGA
Gapdh	TGGAGAAACCTGCCAAGTATG	GGAGACAACCTGGTCCTCAG
