# Supplementary material for: Predicted mouse peroxisome-targeted proteins and their actual subcellular locations
Source: BMC Bioinformatics. 2008 Dec 12;9(Suppl 12):S16. doi: 10.1186/1471-2105-9-S12-S16 (PMC2638156; doi:10.1186/1471-2105-9-S12-S16)
Supplement: Additional file 4 — Details of regular expression and HMMER profile search-derived mouse PTS2 candidates after triage and annotation plus comparison with PeroxisomeDB PTS2 Predictor Blimps Search. [file 1471-2105-9-S12-S16-S4.pdf]

Additional file 4 - Details of regular expression and HMMER profile search-derived mouse PTS2 candidates after triage and annotation plus comparison with PeroxisomeDB PTS2 Predictor Blimps Search

Gene Symbol Accession	Predicted PTS2 Signal	Prediction Method	InterPro motifs/domains	SOSUI TMAP	Selected for Testing	PeroxisomeDB PTS2 Predictor Blimps Search
Galk2 AK082521	RVNIIGEHI 32-40	HMMER E=0.0045	IPR000705 Galactokinase, IPR006203 GHMP kinase, ATP-binding region, IPR006206 Mevalonate and galactokinase, IPR013750 GHMP kinase, C-terminal, IPR014721 Ribosomal protein S5 domain 2-type fold	Soluble 1 TM	Yes(1)	RVNIIGEHI 32-40; E=0.029
Qpctl AK035587	KLRLVVGQL 81-89	HMMER E=0.0091	IPR007484 Peptidase M28	Soluble 1 TM	Yes	KLRLVVGQL 81-89; E=0.013
Sytl3 AB057758	KLKSHLQHL 38-46	HMMER E=0.0059 RE	IPR000008 C2 calcium-dependent membrane targeting, IPR010911 Rab-binding, IPR011011 Zinc finger, FYVE/PHD-type	Soluble 0 TM	Yes	RVRKLKSHL 35-43; E=0.00046
Fut8 AB025198	RVRVLEEQL 82-90	HMMER E=0.0037	IPR001452 Src homology-3	Soluble 1 TM	Yes	RVRVLEEQL 82-90; E=0.0055
Ppp3ca AK150393	RVDILKAHL 42-50	HMMER E=0.0048 RE	IPR004843 Metallophosphoesterase, IPR006186 Serine/threonine-specific protein phosphatase and bis(5-nucleosyl)-tetraphosphatase	Soluble 0 TM	Yes(2)	RVDILKAHL 42-50; E=0.00055
Ppp3cb AK135203	RVDVLKNHL 6-14	HMMER E=0.0089 RE	IPR004843 Metallophosphoesterase, IPR006186 Serine/threonine-specific protein phosphatase and bis(5-nucleosyl)-tetraphosphatase	Soluble 0 TM	No(3)	RVDVLKNHL 6-14; E= 0.00038

Gene Symbol Accession	Predicted PTS2 Signal	Prediction Method	InterPro motifs/domains	SOSUI TMAP	Selected for Testing	PeroxisomeDB PTS2 Predictor Blimps Search
Zmiz1 BC058646	RLQCIKQHL 14-22	HMMER E=0.0016 RE	IPR004181 Zinc finger, MIZ-type	Soluble 0 TM	No(4)	RLQCLKQHL 14-22; E=1.2e-05
Armc6 BC043070	RLQEVSAHL 81-90	HMMER E=0.0016 RE	IPR000225 Armadillo repeat, IPR011989 Armadillo-like helical	Soluble	No(5)	RLQEVSAHL 81-90; E= 3e-05
Wdr6 AK037181	RVQNLLGHF 55-63	HMMER E=0.0037 RE	IPR001680 WD-40 repeat, IPR011046 WD40-like	Soluble 0 TM	No(5)	RVQNLLGHF 55-63; E=0.00017
Adhfe1 AK038853	RVTHLLRHL 8-16	HMMER RE	IPR001670 Iron-containing alcohol dehydrogenase	Soluble 0 TM	No(5)	RVTHLLRHL 8-16; E= 0.00069
Pgm2 BC086490	KIVTVKTQA 3-11	RE	IPR005841 Phosphoglucomutase/ phosphomannomutase, IPR005843 Phosphoglucomutase/phosphomannomutase C-terminal, IPR005844 Phosphoglucomutase/ phosphomannomutase alpha/beta/alpha domain I, IPR005845 Phosphoglucomutase/ phosphomannomutase alpha/beta/alpha domain II, IPR005846 Phosphoglucomutase/ phosphomannomutase alpha/beta/alpha domain III	Soluble 0 TM	No(5)	RLVIGQNGGI 85-93; E= 1.9

Gene Symbol Accession	Predicted PTS2 Signal and Position	Prediction Method	InterPro motifs/domains	SOSUI TMAP	Selected for Testing	PeroxisomeDB PTS2 Predictor Blimps Search
E330021D16Rik AK054382	RLRIVSWHL 22-30	HMMER E=0.0051 RE	IPR000608 Ubiquitin-conjugating enzyme, E2	Soluble 0 TM	No(6)	RLRIVSWHL 22-20; E= 1.8e-05
6030452D12Rik AK031558	RLRVIREQL 29-37	HMMER 0.0021	IPR007733 Agouti	Soluble 0 TM	No(6)	RLRVIREQL 29-37; E=0.0023
2410005O16Rik BC025158	KVEEILAQA 77-85	RE	IPR011990 Tetratricopeptide-like helical	Soluble 0 TM	No(6)	RLWRRLPPL 6-14; E=0.74

Abbreviations. HMMER: Hidden Markov Model profile search; E: E-value of HMMER search result (threshold E＜0.01); RE: regular expression search with perfect match to motif; TM: transmembrane domain. Table rows with a grey background indicate conservation of the PTS2 signal in human orthologs of Galk2, Qpctl, Sytl3, and Zmiz1 or human and rat orthologs of Fut8, Ppp3ca, Ppp3cb and 2410005O16Rik. PTS2 Target Signal Predictor Blimps searches using the first 100 amino acids of the candidate sequences yielded the same putative PTS2 signals as the HMMER searches. Blimps search E-values are shown for comparison purposes, but were not used in selection process for experimental validation. The PTS2 Predictor position-specific weight matrix includes *Arabidopsis* and *Kluyveromyces* (yeast) PTS2 sequences, which were not used for the HMM model, because this study was restricted to rodent and primate sequences.

[(1)]Galk2 is a member of the galactokinase, homoserine, mevalonate, and phosphomevalonate (GHMP) kinase family. The kinase specifically phosporilates N-Acetylgalactosamine (GalNAc) [1], which is also a component in glycosphingolipid biosynthesis. Since peroxisome defective CHO-K1 Z65 [2] cells accumulate glycosphingolipids as seen in the neuronal pathology Zellweger syndrome [3] Galk2 was thought to be along Mvk [4-7] and Mvap [5,8] which are involved in cholesterol synthesis, the third member of GMHP kinases with roles in peroxisomes. Besides, one of its co-expressed genes in Mouse Gene Prediction Database [9] is Pxmp3 (peroxisomal membrane protein 3).

[(2)]The catalytic subunits of calcineurin (PPP3CB and PPP3CA) are known to bind to or co-purify with AKAP5 (AKAP 79) and MAP2 [10,11]. Both proteins are members of the A-kinase anchoring protein family which are implicated as adaptor proteins in co-locating cAMP-dependent protein kinases and phosphatases to various subcellular localizations [12,13]. Since rat A-kinase anchoring protein Akap11 (AKAP 220) was shown to co-localize with an integral peroxisomal membrane protein to a subset of testis peroxisomes [13] Ppp3ca appeared to be an attractive candidate for calcineurin-mediated dephosphorylation of peroxisomal proteins.

[(3)] Ppp3cb was not selected in this study because it encodes only a different catalytic subunit of calcineurin.

[(4)]Zmiz1 was considered of low priority and not selected for testing because its human ortholog co-localizes with AR and SUMO-1 in the nucleus [14].

[(5)]Armc6, Wdr6, Adhfe1, and Pgm2 were not chosen for testing because the predicted PTS2 signal was absent in human orthologs.

[(6)]Problematic clones were not selected for testing. We could not express the E330021D16Rik protein (data not shown). Later its protein record NP_780736 was removed from Refseq and annotated as pseudogene. 6030452D12Rik protein sequence is not supported in RefSeq NM_177904 and mammalian orthologs are unknown; *2410005O16Rik* shows 14 bp differences to the genome sequence, including two amino acid changes in the putative translation.

References

1. Thoden JB and Holden HM: **The molecular architecture of human N-acetylgalactosamine kinase**. *J Biol Chem.* 2005, 280: 32784-32791.
2. Saito M, Iwamori M, Lin B, Oka A, Fujiki Y, Shimozawa N, Kamoshita S, Yanagisawa M, Sakakihara Y: **Accumulation of glycolipids in mutant Chinese hamster ovary cells (Z65) with defective peroxisomal assembly and comparison of the metabolic rate of glycosphingolipids between Z65 cells and wild-type CHO-K1 cells**. *Biochim Biophys Acta* 1999, 1438: 55-62.
3. Pettus BJ, Baes M, Busman M, Hannun YA, Van Veldhoven PP: **Mass spectrometric analysis of ceramide perturbations in brain and fibroblasts of mice and human patients with peroxisomal disorders**. *Rapid Commun Mass Spectrom*. 2004, 18: 1569-1574.
4. Biardi L, Sreedhar A, Zokaei A, Vartak NB, Bozeat RL, Shackelford JE, Keller GA, Krisans SK: **Mevalonate kinase is predominantly localized in peroxisomes and is defective in patients with peroxisome deficiency disorders**. *J Biol Chem.* 1994, 269: 1197-1205.
5. Olivier LM, Chambliss, KL, Gibson KM, Krisans SK: **Characterization of phosphomevalonate kinase: chromosomal localization, regulation, and subcellular targeting**. *J Lipid Res*. 1999, 40: 672-679.
6. Hogenboom S, Tuyp JJ, Espeel M, Koster J, Wanders RJ, Waterham HR: **Mevalonate kinase is a cytosolic enzyme in humans**. *J Cell Sci.* 2004, 117: 631-639.
7. Kovacs WJ, Tape KN, Shackelford JE, Duan X, Kasumov T, Kelleher JK, Brunengraber H, Krisans SK: **Localization of the pre-squalene segment of the isoprenoid biosynthetic pathway in mammalian peroxisomes**. *Histochem Cell Biol*. 2007, 127: 273-290.
8. Hogenboom S, Tuyp JJ, Espeel M, Koster J, Wanders RJ, Waterham HR: **Phosphomevalonate kinase is a cytosolic protein in humans**. *J Lipid Res*. 2004, 45: 697-705.

9. Zhang W, Morris QD, Chang R, Shai O, Bakowski MA, Mitsakakis N, Mohammad N, Robinson MD, Zirngibl R, Somogyi E, et al.: **The functional landscape of mouse gene expression**. *J Biol.* 2004, 3: 21.

10. Coghlan VM, Perrino BA, Howard M, Langeberg LK, Hicks JB, Gallatin WM, Scott JD: **Association of protein kinase A and protein phosphatase 2B with a common anchoring protein**. *Science* 1995, 267: 108-111.

11. Nunbhakdi-Craig V, Machleidt T, Ogris E, Bellotto D, White CL 3rd, Sontag E: **Protein phosphatase 2A associates with and regulates atypical PKC and the epithelial tight junction complex**. *J Cell Biol.* 2002, 158: 967-978.

12. Lester LB, Coghlan VM, Nauert B, Scott JD: **Cloning and characterization of a novel A-kinase anchoring protein. AKAP 220, association with testicular peroxisomes**. *J Biol Chem*. 1996, 271: 9460-9465.

13. Feliciello A, Gottesman ME, Avvedimento EV: **The biological functions of A-kinase anchor proteins**. *J Mol Biol*. 2001, 308: 99-114.

14. Sharma M, Li X, Wang Y, Zarnegar M, Huang CY, Palvimo JJ, Lim B, Sun Z: **hZimp10 is an androgen receptor co-activator and forms a complex with SUMO-1 at replication foci**. *EMBO J*. 2003, 22: 6101-6114.
