# Supplementary material for: Predicted mouse peroxisome-targeted proteins and their actual subcellular locations
Source: BMC Bioinformatics. 2008 Dec 12;9(Suppl 12):S16. doi: 10.1186/1471-2105-9-S12-S16 (PMC2638156; doi:10.1186/1471-2105-9-S12-S16)

## Additional file 5 – CHO-perRed after two and five passages

DsRed2 expression is not affected by cell passages of CHO-perRed. Left side: Bright field images (original magnification, 200 x). Right side: fluorescent microscopy images of CHO-perRed cells (original magnification, 200 x).

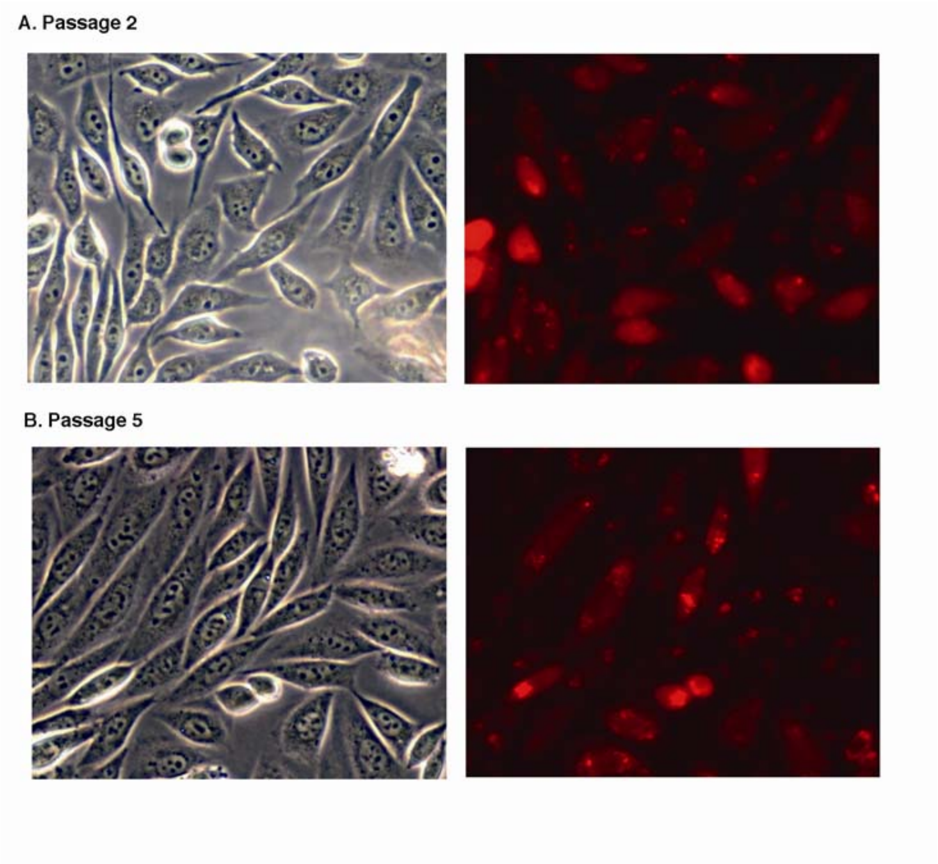

Supplement: Additional file 5 — CHO-perRed after two and five passages. [file 1471-2105-9-S12-S16-S5.pdf]
