# Supplementary material for: Predicted mouse peroxisome-targeted proteins and their actual subcellular locations
Source: BMC Bioinformatics. 2008 Dec 12;9(Suppl 12):S16. doi: 10.1186/1471-2105-9-S12-S16 (PMC2638156; doi:10.1186/1471-2105-9-S12-S16)

Additional File 6 – Measurement of Zadh2 liver mRNA levels by using quantitative real-time PCR

N: control mice fed with standard rodent chow (n=3/group), B: bezafibrate treated mice (n=3/group), H; mice fed with high-fat diet (n=3/group). Data were normalized against Gapdh expression levels and reported as mean values + SD.Expression of Zadh2 upon bezafibrate treatment. Thiolase and Scp were included as positive and negative controls for bezafibrate-induced expression.

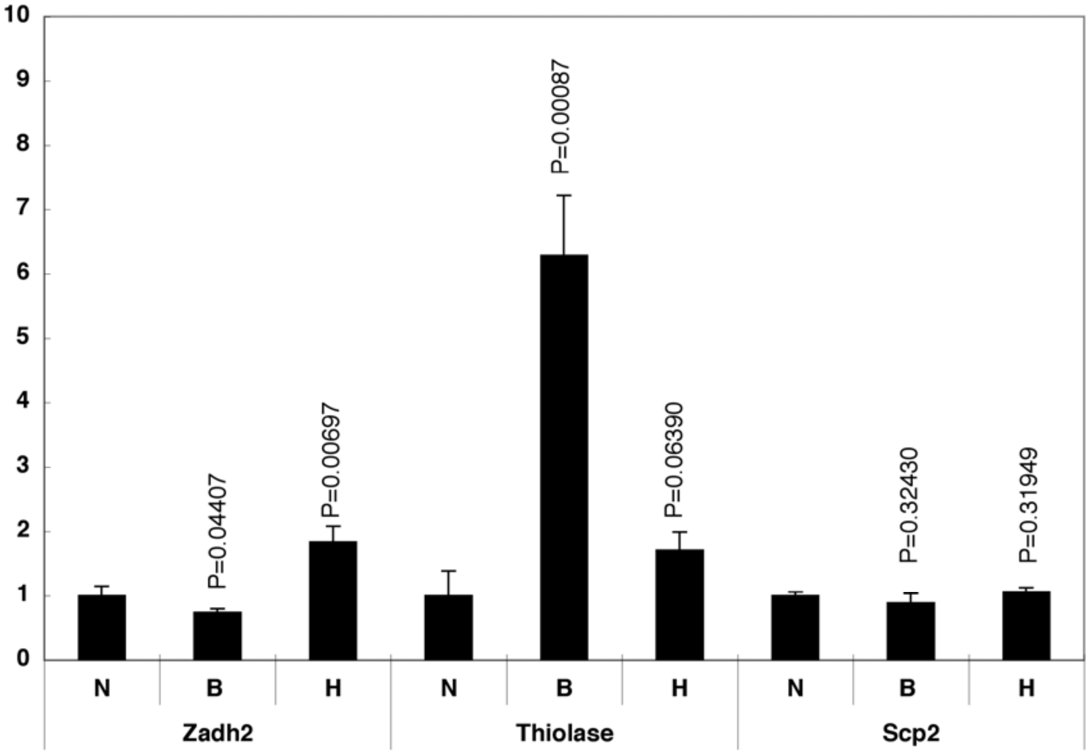

Supplement: Additional file 6 — Measurement of Zadh2 liver mRNA levels by using quantitative real-time PCR. [file 1471-2105-9-S12-S16-S6.pdf]
