# Supplementary material for: Predicted mouse peroxisome-targeted proteins and their actual subcellular locations
Source: BMC Bioinformatics. 2008 Dec 12;9(Suppl 12):S16. doi: 10.1186/1471-2105-9-S12-S16 (PMC2638156; doi:10.1186/1471-2105-9-S12-S16)
Supplement: Additional file 7 — Non-peroxisomal localization of five PTS2- and one predicted PTS1-containing candidates in CHO-perRed cells. [file 1471-2105-9-S12-S16-S7.pdf]

Additional File 7 - Non-peroxisomal localization of five PTS2- and one predicted PTS1-containing candidates in CHO-perRed cells

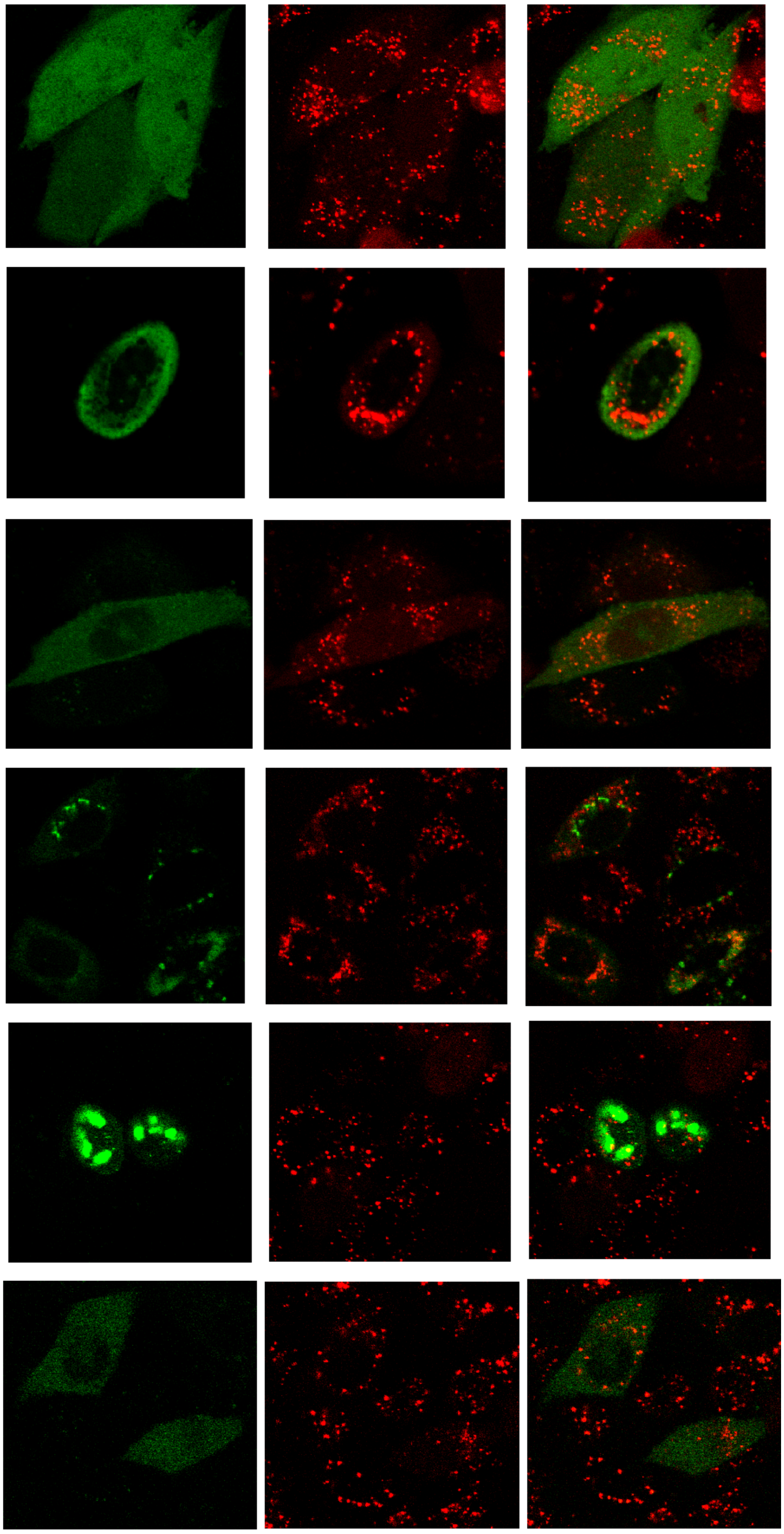

original magnification, 630 x
