# Supplementary material for: Predicted mouse peroxisome-targeted proteins and their actual subcellular locations
Source: BMC Bioinformatics. 2008 Dec 12;9(Suppl 12):S16. doi: 10.1186/1471-2105-9-S12-S16 (PMC2638156; doi:10.1186/1471-2105-9-S12-S16)
Supplement: Additional file 8 — Comparison of predicted subcellular locations with experimental data for both known and hitherto unknown PTS1 and PTS2-targeted proteins. A. Comparison of LOCATE summaries and integrated prediction results with experimentally supported localization of proteins. B. Comparison of PTS1Prowler/PProwler and PTS1 Predictors predictions with experimentally supported localization of proteins. [file 1471-2105-9-S12-S16-S8.pdf]

Additional file 8 – Comparison of predicted subcellular locations with experimental data for both known and hitherto unknown PTS1 and PTS2-targeted proteins

A. Comparison of LOCATE summaries and integrated prediction results with experimentally supported localization of proteins

**Protein**	**LOCATE SCL Summary**	**Individual subcellular location (SCL) predictions intergrated in LOCATE**										**Experimentally supported localization (this study or from literature)**
		**CELLO**		**pTarget**		**Proteome Analyst**		**WoLF PSORT**		**MultiLoc**		
**PTS1-containing proteins with hitherto unknown peroxisomal localization**												
KBTBD 10 [1]	**PM, Cyt, Chs** (DB); **soluble, non-secreted**	**Cyt**	2.94	**Cyt**	87.60	**Cyt**<br>Nuc<br>ER	1.00<br>0.00<br>0.00	**Cyt**<br>Nuc<br>Per	23.00<br>4.00<br>2.00	**Per**<br>Cyt<br>ER	0.80<br>0.15<br>0.02	**Cyt** [this study], **Cyt**, PM (pseudopodia) of FBR fibroblast cell line [2,3]
Gab2 [1]	**No SCL data; soluble, non-secreted**	**Nuc**	4.50	**Nuc**	100.00	**Cyt**<br>Nuc<br>ExR	0.92<br>0.00<br>0.00	**Nuc**<br>Cyt-Nuc	28.50<br>16.50	**Nuc**<br>Per<br>Cyt	0.88<br>0.09<br>0.02	**Cyt**, **PM**, **End** of EGF-induced hepatocytes [4]
Scrab1 [1]	**No SCL data; multipass-membrane**	**PM**	2.24	**Lys**	81.40	**PM**<br>ER<br>Gol	0.93<br>0.01<br>0.00	**ER**<br>PM<br>Per	16.00<br>9.00<br>4.00	**ExR**<br>ER<br>Lys	0.77<br>0.11<br>0.08	**PM** (caveolae) of endothelial cells [5]
**Recently identified PTS1-containing proteins with known peroxisomal localization**												
Zadh2 [1]	**No SCL data; soluble, non-secreted**	**Mit**<br>Chl	1.85<br>1.36	**Mit**	87.60	**Cyt**<br>ER<br>Mit	0.99<br>0.00<br>0.00	**Cyt**<br>Cyt-Nuc<br>Mit	14.00<br>11.00<br>7.00	**Mit**<br>Per<br>Cyt	0.71<br>0.27<br>0.01	**Per** [this study], **Per** of CHO cells and rat liver peroxisomal fraction [6,7]
Tysnd1 [1]	**No SCL data; soluble, non-secreted**	**PM**	2.44	**Lys**	100.00	**ExR**<br>Cyt<br>ER	0.98<br>0.00<br>0.00	**Mit**<br>Cyt-Nuc<br>Cyt	14.00<br>7.33<br>7.00	**PM**<br>**Per**<br>Mit	0.41<br>0.30<br>0.11	**Per** [6,7] of CHO cells and rat liver peroxisomal fraction

Protein	LOCATE SCL Summary	Individual subcellular location (SCL) predictions intergrated in LOCATE										Experimentally supported localization (this study or from literature)
		CELLO		pTarget		Proteome Analyst		WoLF PSORT		MultiLoc*		
**PTS2-containing proteins with hitherto unknown peroxisomal localization**												
Galk2	**No SCL data; soluble, non-secreted**	**Cyt**	1.92	**Cyt**	100.00	**Cyt**<br>ER<br>Nuc	0.86<br>0.00<br>0.00	**Cyt**<br>Cyt-Mit<br>PM	14.50<br>9.00<br>7.00	**Cyt**<br>PM<br>Per	0.98<br>0.00<br>0.00	**Cyt** [this study]
Qpctl	**No SCL data; multipass-membrane**	**PM**<br>Mit	1.37<br>1.34	**PM**	50.10	ExR<br>Cyt<br>ER	0.03<br>0.00<br>0.00	**ER**<br>Per<br>Cyt-Nuc	6.00<br>5.50<br>5.00	ER<br>Gol<br>PM	0.39<br>0.36<br>0.06	**Cyt** [this study]
Sytl3	**No SCL data; soluble, non-secreted**	**Nuc**	3.87	**Cyt**	87.60	**Cyt**<br>PM<br>Nuc	1.00<br>0.00<br>0.00	**Nuc**<br>Cyt-Nuc<br>Cyt	18.00<br>17.00<br>14.00	**Cyt**<br>Mit<br>PM	0.84<br>0.05<br>0.03	**Cyt** [this study], **Dcv** of pancreatic beta cell lines (beta HC9 and MIN6) [8] and neuronal PC12 cell line [9]
Fut8	**Gol** (Lit)**; type II membrane**	**Cyt**	2.59	**Gol**	93.90	**Gol**<br>ER<br>Cyt	1.00<br>0.27<br>0.00	**ER**<br>ER-Gol<br>ExR	11.50<br>8.50<br>7.00	**Gol**<br>ER<br>Lys	0.83<br>0.09<br>0.03	**Cyt structures in proximity to Nuc** [this study], **Gol** (Golgi-rich fraction of rat liver) [10]
Ppp3ca	**Cyt; Nuc** (Lit); **soluble, non-secreted**	**Cyt**	2.59	**Cyt**	87.60	**Cyt**<br>ER<br>Nuc	0.99<br>0.00<br>0.00	**Cyt**<br>Cyt_PM<br>Nuc	20.50<br>11.00<br>5.00	**Cyt**<br>Per<br>PM	0.96<br>0.01<br>0.01	**Cyt** and **Nuc** of skeletal muscle, COS7 cell line [11], and aortic myocytes [12] ; translocation from **Cyt** to **Nuc** in keratinocytes upon intracellular Ca^{2+} increase [13]; mainly **Cyt**; minority **Nuc** [this study]

**Protein**	**LOCATE SCL Summary**	**Individual subcellular location (SCL) predictions intergrated in LOCATE**										**Experimentally supported localization (this study or from literature)**
		**CELLO**		**pTarget**		**Proteome Analyst**		**WoLF PSORT**		**MultiLoc***		
**PTS2-containing proteins with hitherto unknown peroxisomal localization**												
Ppp3cb	**Cyt; Nuc** (Lit); **soluble, non-secreted**	**PM**<br>**Nuc**	1.61<br>1.07	**Cyt**	56.30	**Cyt**<br>ER<br>Nuc	0.99<br>0.00<br>0.00	**Cyt**<br>**Cyt-Nuc**<br>Nuc	9.50<br>8.50<br>6.50	**Cyt**<br>PM<br>Per	0.92<br>0.04<br>0.02	**Cyt** aortic myocytes [12]; upon PDGF induction translocalization from **Cyt** to **Nuc** in aortic myocytes [12], translocation from **Cyt** to **Nuc** in keratinocytes upon intracellular Ca^{2+} increase [13]
Zmiz1	**Cyt** (DB); **soluble, non-secreted**	**Cyt**	4.11	**Cyt**	100.00	**Nuc**<br>Cyt<br>ER	1.00<br>0.00<br>0.00	**Nuc**<br>**Cyt**<br>PM	13.00<br>9.00<br>5.00	**Nuc**<br>Cyt<br>Per	0.96<br>0.02<br>0.01	**Nuc** in monkey kidney cell line CV-1, prostate cancer cell line PC13 [14]
Wdr6	**No SCL data; soluble, non-secreted**	**Cyt**	3.04	**Cyt**	75.10	**Cyt**<br>Nuc<br>ER	0.99<br>0.02<br>0.00	**Cyt**<br>Nuc<br>ExR	19.00<br>8.00<br>2.00	**Cyt**<br>Per<br>ER	0.82<br>0.10<br>0.05	**Cyt** in embryo kidney cell line HEK293T [15]
Adhfe1	**No SCL data; soluble, non-secreted**	**Mit**	2.33	**Mit**	93.90	**Cyt**<br>Mit<br>ER	0.37<br>0.00<br>0.00	**Mit**	29.00	**Mit**<br>ExR<br>Per	0.99<br>0.01<br>0.00	**Mit** in COS cells [16]
Pgm2	**No SCL data; soluble, non-secreted**	**Cyt**	2.42	**Cyt**	81.40	**Cyt**<br>ER<br>Gol	1.00<br>0.79<br>0.00	**Cyt**<br>**Mit**	21.00<br>11.00	**Cyt**<br>**Per**<br>Mit	0.69<br>0.26<br>0.03	**Cyt;** mouse liver cytosolic fraction; identified by MALD-TOF mass spectrometry [17]

Protein	LOCATE SCL Summary	Individual subcellular location (SCL) predictions intergrated in LOCATE										Experimentally supported localization (this study or from literature)
		CELLO		pTarget		Proteome Analyst		WoLF PSORT		MultiLoc*		
**PTS2-containing proteins with known peroxisomal localization**												
Pex11c	**Per** (DB); **CP** (Exp.;Hela cells) **Multipassmem.**	**PM**	2.33	**Per**	100.0	NA	NA	**Mit**<br>Cyt-Nuc<br>Mit-Per	16.00<br>8.33<br>8.33	**Mit**<br>Per<br>Cyt	0.51<br>0.26<br>0.12	**Per** in fibroblasts [18]
Phyh	**Per** (DB); **soluble, non-secreted**	**Per**	2.99	**Per**	50.10	**Per**<br>ER<br>Cyt	0.99<br>0.00<br>0.00	**Mit**<br>Cyt	27.00<br>4.00	**Mit**<br>Cyt<br>ER	0.71<br>0.09<br>0.08	**Per**; liver subcellular fraction [19]
Acaa1	**Per** (Lit; DB); **soluble, non-secreted**	**Per**<br>Chl	1.47<br>1.26	**Per**	68.90	**Mit**<br>Per<br>Cyt	0.35<br>0.00<br>0.00	**Mit**<br>Nuc<br>Cyt-Nuc	13.00<br>7.50<br>7.50	**Mit**<br>Per<br>Cyt	0.98<br>0.01<br>0.00	**Per**; liver subcellular fraction [20] and CVH-Px110 cell line (derived from CV-1) [21]
Acaa1b	**No SCL data; soluble, non-secreted**	**Per**	2.04	**Lys**	81.40	**Mit**<br>Per<br>Cyt	0.35<br>0.00<br>0.00	**Mit**<br>Cyt-Nuc<br>Cyt	11.00<br>8.50<br>7.50	**Mit**<br>Per<br>Cyt	0.98<br>0.01<br>0.00	**Per** in CVH-Px110 cell line [21]
Mvk	**Cyt, Per** (DB); **soluble, non-secreted**	**Cyt**	1.82	**Cyt**	1.00	**Cyt**<br>Per<br>ER	0.99<br>0.38<br>0.01	**ExR**<br>Mit<br>ER	11.00<br>8.50<br>5.00	**Cyt**<br>ExR<br>Mit	0.42<br>0.27<br>0.16	**Per** in CV1 cells, skin fibroblasts, liver peroxisomal subfraction; **Cyt** and **Per** in liver by electron microscopy [22], **Cyt** in Hek293, CV1 and fibroblasts subcellular fractions [23], **Per** in hepatoblastoma HepG2 cell line [24]

Protein	LOCATE SCL Summary	Individual subcellular location (SCL) predictions intergrated in LOCATE										Experimentally supported localization (this study or from literature)
		CELLO		pTarget		Proteome Analyst		WoLF PSORT		MultiLoc*		
**PTS2-containing proteins with hitherto unknown peroxisomal localization**												
Agps	**Per** (DB);<br>**soluble, non-secreted**	**Per**	2.21	**Per**	87.60	**Per**<br>ER<br>Mit	1.00<br>0.20<br>0.15	**Cyt**<br>Cyt-Nuc<br>Cyt-PM	19.50<br>14.83<br>11.16	**Cyt**<br>Per<br>Mit	0.79<br>0.17<br>0.03	**Per**; liver peroxisomal subfraction [25]

For each protein the integrated LOCATE result derived from five subcellular localization prediction methods is shown. The predicted localization is accompanied by a score that is specific for the method. Details are described in the FAQ of LOCATE (http://locate.imb.uq.edu.au/). Abbreviations. DB: database annotation; Lit: Literature; Exp: experimental; Cyt: cytoplasma; CP: ctyoplasmic puncta; Dcv: dense core vesicles; Nuc: nucleus; Per: peroxisomes; Mit: mitochondria: End: endosomes; ER: endoplasmatic reticulum; Lys: lysosome; PL: plasma membrane; ExR: extracellular region; Chl: chlorplast; Chs: cytoskeleton; Gol: Golgi apparatus; NA: not assigned; *MultiLoc was not trained to detect PTS2 signals; Gab2, Scrab1, and KBTBD10 are candidates that were identified in a previous study [1]. The localization of Sytl3, an effector of Rab27a-dependent membrane trafficking [26] the observed localization appears to depend on the ontogeny of the cells. The localization to dense core vesicles cannot be predicted by any of the programs as they are not trained for this location.

The LOCATE accessions and their corresponding GenPept or ENSEMBL accessions are: KBTBD10: hPA13085.1, NP_006054; Gab2: mPA18842.1, AAD05166; Scrab1: mPA17109.1, AAH04656; Zadh2: mPA10238.1, BAC38389; Tysnd1: mPA1614.1, BAC38389; Galk2: mPA11864.1,

AAH79843; Qpctl: mPA19634.1, BAC29114; Sytl3: mPA8872.1, BAB41086; Fut8: mPA4396.3, BAE22175 ; Ppp3ca: mPA13948.1, ENSMUSP00000053101; Ppp3cb: mPA6713.2, AAH66000; Zmiz1: mPA6589.1, AAH65120; Wdr6: mPA22875.1, NP_001020546; Adhfe1: mPA421.1, BAC30151; Pgm2: mPA14944.1; BAE28266; Pex11c: mPA21256.1, AAH62135; Phyh: mPA11499.1; AAH02018; Acaa1: mPA22546.1, AAP31668; Acaa1b: mPA23310.1, ENSMUSP00000010795; Mvk: mPA16137.2, AAH05606; Agps: mPA12066.1, AAH63086.

Selection criteria. All known mammalian PTS2-targeted proteins are represented by six mouse sequences. The other sequences represent ten of 13 PTS2-targeted candidates that were predicted in this study (see also Additional File 4). Three candidates were excluded from the comparison because of problems with the original cDNA clone or its sequence (see Additional File 4; Footnote 6). Five of the ten candidates were shown to locate in this and other studies to non-peroxisomal locations. The other five candidates were not experimentally tested by us (low priority). Therefore the comparison of the predicted location relies only on experiments reported in the literature.

B. Comparison of PTS1Prowler/PProwler and PTS1 Predictors predictions with experimentally supported localization of proteins

Protein	GenPept Accession	PTS1 Prowler/ PProwler		PTS1 Predictor		PeroxisomeDB PTS1 Predictor (Blimps Search)		Experimentally supported localization (this study or from literature)
**PTS1-containing proteins with hitherto unknown peroxisomal localization**								
KBTBD10 [1]	NP_006054	Per<br>Oth<br>**Mtp**<br>Sp	0.02<br>0.02<br>0.96<br>0.00	Per	2.052<br>*P* FP 0.14%	**NA**	E-value 0.32	**Cyt** [this study], **Cyt**, **PM** (pseudopodia) of FBR fibroblast cell line [2,3]
Gab2 [1]	AAD05166	**Per**<br>**Oth**<br>Mtp<br>Sp	0.46<br>0.96<br>0.02<br>0.01	**Per**	8.628<br>*P* FP 1.7e-04	**NA**	E-value 0.12	**Cyt**, **PM**, **End** of EGF-induced hepatocytes [4]
Scrab1 [1]	AAH04656	Per<br>Oth<br>Mtp<br>**Sp**	0.05<br>0.10<br>0.01<br>0.90	Per	3.627<br>*P* FP 8.8e-04	**NA**	E-value 1	**PM** (caveolae) of endothelial cells [5]
**Recently identified PTS1-containing proteins with known peroxisomal localization**								
Zadh2 [1]	BAC38389	**Per**<br>Oth<br>**Mtp**<br>Sp	0.99<br>0.24<br>0.76<br>0.00	Per	2.734<br>*P* FP 0.11%	**NA**	E-value 0.0063	**Per** [this study], Per of CHO cells and rat liver peroxisomal fraction [6,7]

Protein	GenPept Accession	PTS1 Prowler/ PProwler		PTS1 Predictor		PeroxisomeDB PTS1 Predictor (BlimpsSearch)		Experimentally supported localization (this study or from literature)
**Recently identified PTS1-containing proteins with known peroxisomal localization**								
Tysnd1 [1]	BAB23793	**Per**<br>**Oth**<br>Mtp<br>Sp	0.98<br>0.96<br>0.04<br>0.00	**Per**	16.264<br>*P* FP 5.4e-06	**NA**	E-value<br>0.045	**Per** [6,7] of CHO cells and rat liver peroxisomal fraction

Selection criteria: Four mouse (Gab2, Scrab1, Zadh1, and Tsynd1) proteins and human KBTBD10 were previously predicted by us as strong PTS1-targeted candidates [1]. Two of the candidates, Tysnd1 [6; 7] and Zadh [this study; 7] were experimentally confirmed by us [6] and other [7] as PTS1-targeted. Abbreviations. Per: peroxisome; Oth: others; Mtp: mitochondrial protein; Sp: secreted protein. *P* FP: probability of false positive; NA: not applicable; Blimps provides all possible hits over the entire sequence length, together with alignment scores and E-values based on the position-specific scoring matrix of a twelve amino acids PTS1 profile. The decision making whether the protein of interest might be targeted to peroxisomes is left to the user.

References

1. Kurochkin IV, Nagashima T, Konagaya A, Schönbach C: **Sequence-based discovery of the human and rodent peroxisomal proteome**. *Appl. Bioinformatics*. 2005, 4: 93-104.
2. Spence HJ, Johnston I, Ewart K, Buchanan SJ, Fitzgerald U, Ozanne BW: **Krp1, a novel kelch related protein that is involved in pseudopod elongation in transformed cells**. *Oncogene* 2000, 19: 1266-1276.

3. Spence HJ, McGarry L, Chew CS, Carragher NO, Scott-Carragher LA, Yuan Z, Croft DR, Olson MF, Frame M, Ozanne BW: **AP-1 differentially expressed proteins Krp1 and fibronectin cooperatively enhance Rho-ROCK-independent mesenchymal invasion by altering the function, localization, and activity of nondifferentially expressed proteins**. *Mol Cell Biol.* 2006, 26: 1480-1495.
4. Kong M, Mounier C, Balbis A, Baquiran G, Posner BI: **Gab2 tyrosine phosphorylation by a pleckstrin homology domain-independent mechanism: role in epidermal growth factor-induced mitogenesis**. *Mol Endocrinol*. 2003, 17: 935-944.
5. Uittenbogaard A, Shaul PW, Yuhanna IS, Blair A, Smart EJ: **High density lipoprotein prevents oxidized low density lipoprotein-induced inhibition of endothelial nitric-oxide synthase localization and activation in caveolae**. *J Biol Chem*. 2000, 275: 11278-11283.
6. Kurochkin IV, Mizuno Y, Konagaya A, Sakaki Y, Schönbach C, Okazaki Y: **Novel peroxisomal protease Tysnd1 processes PTS1- and PTS2-containing enzymes involved in beta-oxidation of fatty acids**. *EMBO J* 2007, 26: 835-845.
7. Islinger M, Luers GH, Li KW, Loos M, Volkl A: **Rat Liver Peroxisomes after Fibrate Treatment: A survey using quantitative Mass spectrometry**. *J Biol Chem*. 2007, 282: 23055-23069.
8. Wang J, Takeuchi T, Yokota H, Izumi T: **Novel rabphilin-3-like protein associates with insulin-containing granules in pancreatic beta cells**. *J Biol Chem.* 1999, 274: 28542-28548.
9. Fukuda M, Kanno E, Saegusa C, Ogata Y, Kuroda TS: **Slp4-a/granuphilin-a regulates dense-core vesicle exocytosis in PC12 cells**. *J Biol Chem*. 2002, 277: 39673-39678.

10. Longmore GD and Schachter H: **Product-identification and substrate-specificity studies of the GDP-L-fucose:2-acetamido-2-deoxy-beta-D-glucoside (FUC goes to Asn-linked GlcNAc) 6-alpha-L-fucosyltransferase in a Golgi-rich fraction from porcine liver**. *Carbohydr Res.* 1982, 100: 365-392.

11. Torgan CE and Daniels MP: **Calcineurin localization in skeletal muscle offers insights into potential new targets**. *J Histochem Cytochem*. 2006, 54: 119-128.

12. Jabr RI, Wilson AJ, Riddervold MH, Jenkins AH, Perrino BA, Clapp LH. Nuclear translocation of calcineurin Abeta but not calcineurin Aalpha by platelet-derived growth factor in rat aortic smooth muscle. *Am J Physiol Cell Physiol.* 2007, 292: C2213-2225.

13. Al-Daraji WI, Grant KR, Ryan K, Saxton A, Reynolds NJ: **Localization of calcineurin/NFAT in human skin and psoriasis and inhibition of calcineurin/NFAT activation in human keratinocytes by cyclosporin A**. *J Invest Dermatol.* 2002, 118: 779-788.

14. Li X, Thyssen G, Beliakoff J, Sun Z: **The novel PIAS-like protein hZimp10 enhances Smad transcriptional activity**. *J Biol Chem.* 2006, 281: 23748-23756.

15. Xie X, Wang Z, Chen Y: **Association of LKB1 with a WD-repeat protein WDR6 is implicated in cell growth arrest and p27(Kip1) induction**. *Mol Cell Biochem*. 2007, 301: 115-122.

16. Kim JY, Tillison KS, Zhou S, Lee JH, Smas CM: **Differentiation-dependent expression of Adhfe1 in adipogenesis**. *Arch Biochem Biophys*. 2007, 464:100-111.

17. Zgoda V, Tikhonova O, Viglinskaya A, Serebriakova M, Lisitsa A, Archakov A: **Proteomic profiles of induced hepatotoxicity at the subcellular level**. Proteomics. 2006, 6:4662-4670.

18. Li X, Baumgart E, Dong GX, Morrell JC, Jimenez-Sanchez G, Valle D, Smith KD and Gould SJ: **PEX11alpha is required for peroxisome proliferation in response to 4-phenylbutyrate but is dispensable for peroxisome proliferator-activated receptor alpha-mediated peroxisome proliferation**. *Mol Cell Biol*. 2002, 22: 8226-8240.

19. Jansen GA, Ofman R, Denis S, Ferdinandusse S, Hogenhout EM, Jakobs C, Wanders RJ: **Phytanoyl-CoA hydroxylase from rat liver. Protein purification and cDNA cloning with implications for the subcellular localization of phytanic acid alpha-oxidation**. *J Lipid Res*. 1999, 40: 2244-2254.

20. Miyazawa S, Osumi T, Hashimoto T: **The presence of a new 3-oxoacyl-CoA thiolase in rat liver peroxisomes**. Eur J Biochem 1980, 103: 589-596.

21. Swinkels BW, Gould SJ, Bodnar AG, Rachubinski RA, Subramani S: **A novel, cleavable peroxisomal targeting signal at the amino-terminus of the rat 3-ketoacyl-CoA thiolase**. *EMBO J*. 1991, 10: 3255-3262.

22. Biardi L, Sreedhar A, Zokaei A, Vartak NB, Bozeat RL, Shackelford JE, Keller GA, Krisans SK: **Mevalonate kinase is predominantly localized in peroxisomes and is defective in patients with peroxisome deficiency disorders**. *J Biol Chem*. 1994, 269: 1197-1205.

23. Hogenboom S, Tuyp JJ, Espeel M, Koster J, Wanders RJ, Waterham HR: **Mevalonate kinase is a cytosolic enzyme in humans**. *J Cell Sci*. 2004, 117: 631-639.

24. Kovacs WJ, Tape KN, Shackelford JE, Duan X, Kasumov T, Kelleher JK, Brunengraber H, Krisans SK: **Localization of the pre-squalene segment of the isoprenoid biosynthetic pathway in mammalian peroxisomes**. *Histochem Cell Biol*. 2007, 127: 273-29

25. Biermann J, and van den Bosch H: **In vitro processing of the human alkyl-dihydroxyacetonephosphate synthase precursor**. *Arch Biochem Biophys*. 1999, 368: 139-146.
26. Kuroda TS, Fukuda M, Ariga H, Mikoshiba K: **The Slp homology domain of synaptotagmin-like proteins 1-4 and Slac2 functions as a novel Rab27A binding domain**. *J Biol Chem*. 2002, 277: 9212-9218.
